# Overexpression of TFAM or Twinkle Increases mtDNA Copy Number and Facilitates Cardioprotection Associated with Limited Mitochondrial Oxidative Stress

**DOI:** 10.1371/journal.pone.0119687

**Published:** 2015-03-30

**Authors:** Masataka Ikeda, Tomomi Ide, Takeo Fujino, Shinobu Arai, Keita Saku, Takamori Kakino, Henna Tyynismaa, Toshihide Yamasaki, Ken-ichi Yamada, Dongchon Kang, Anu Suomalainen, Kenji Sunagawa

**Affiliations:** 1 Department of Cardiovascular Medicine, Graduate School of Medical Sciences, Kyushu University, Fukuoka, Japan; 2 Research Programs Unit, Molecular Neurology, University of Helsinki, Biomedicum Helsinki, Helsinki, Finland; 3 Department of Biofunctional Science, Faculty of Pharmaceutical Sciences, Kyushu University, Fukuoka, Japan; 4 Department of Clinical Chemistry and Laboratory Medicine, Graduate School of Medical Sciences, Kyushu University, Fukuoka, Japan; Rutgers New Jersey Medical School, UNITED STATES

## Abstract

**Background:**

Mitochondrial DNA (mtDNA) copy number decreases in animal and human heart failure (HF), yet its role in cardiomyocytes remains to be elucidated. Thus, we investigated the cardioprotective function of increased mtDNA copy number resulting from the overexpression of human transcription factor A of mitochondria (TFAM) or Twinkle helicase in volume overload (VO)-induced HF.

**Methods and Results:**

Two strains of transgenic (TG) mice, one overexpressing TFAM and the other overexpressing Twinkle helicase, exhibit an approximately 2-fold equivalent increase in mtDNA copy number in heart. These TG mice display similar attenuations in eccentric hypertrophy and improved cardiac function compared to wild-type (WT) mice without any deterioration of mitochondrial enzymatic activities in response to VO, which was accompanied by a reduction in matrix-metalloproteinase (MMP) activity and reactive oxygen species after 8 weeks of VO. Moreover, acute VO-induced MMP-2 and MMP-9 upregulation was also suppressed at 24 h in both TG mice. In isolated rat cardiomyocytes, mitochondrial reactive oxygen species (mitoROS) upregulated MMP-2 and MMP-9 expression, and human TFAM (hTFAM) overexpression suppressed mitoROS and their upregulation. Additionally, mitoROS were equally suppressed in H9c2 rat cardiomyoblasts that overexpress hTFAM or rat Twinkle, both of which exhibit increased mtDNA copy number. Furthermore, mitoROS and mitochondrial protein oxidation from both TG mice were suppressed compared to WT mice.

**Conclusions:**

The overexpression of TFAM or Twinkle results in increased mtDNA copy number and facilitates cardioprotection associated with limited mitochondrial oxidative stress. Our findings suggest that increasing mtDNA copy number could be a useful therapeutic strategy to target mitoROS in HF.

## Introduction

Heart failure (HF) continues to be an unsolved problem in developed countries.[1] During HF, hemodynamic load and neuro-hormonal activation cause structural and functional alterations of the heart, which start initially as adaptive responses, but later become maladaptive, and eventually lead to the vicious cycle of HF.[2] In the myocardium, mitochondria are not only a critical source of adenosine tri-phosphate (ATP) but also produce reactive oxygen species (ROS). Many study groups, including ours, have shown that mitochondrial ROS (mitoROS) elicit mitochondrial DNA (mtDNA) damage and impair mitochondrial electron transport chain (ETC) activity, resulting in additional mitoROS production. Notably, the presence of excess mitoROS is associated with maladaptive cardiac remodeling and HF progression in experimental animal models.[3–7]

Mitochondrial transcription factor A (TFAM), which was initially cloned as an mtDNA transcription factor, also plays an essential role in the maintenance of mtDNA and mitochondrial homeostasis.[8,9] Heart-specific TFAM knockout mice present with a critical depletion in mtDNA copy number, progressive respiratory chain deficiency in the myocardium, and lethality due to severe cardiac dysfunction.[10,11] In contrast, TFAM overexpression increases mtDNA copy number, ameliorates cardiac remodeling, and improves the survival after myocardial infarction (MI) in mice.[12] However, the precise roles and the protective mechanisms of mtDNA quantity in HF remain to be elucidated.

To examine the effect of increased mtDNA copy number on cardioprotection, we used transgenic (TG) mice that overexpress TFAM (TFAM mice) or Twinkle (Twinkle mice). Twinkle is a nuclear DNA-encoded mitochondrial DNA helicase that exhibits structural similarity to T7 phage helicase/primase, and is associated with heritable neuromuscular mitochondrial disease.[13] TFAM increases mtDNA stability and the proportion of non-replicating nucleoids, whereas Twinkle enhances mtDNA replication.[14] Despite acting through different mechanisms, overexpression of either Twinkle or TFAM in mice increases mtDNA copy number in mice.

We recently demonstrated that recombinant human TFAM (rhTFAM) suppresses angiotensin-induced hypertrophy in myocytes;, which have no deterioration of mtDNA transcription or translation[15]; this suggests that TFAM overexpression and/or the associated increase in mtDNA copy number might impart cardioprotective effects on cardiomyocytes in the absence of mtDNA depletion and mitochondrial dysfunction. In our preliminary experiments, involving some HF mouse models, no significant mtDNA depletion and mitochondrial dysfunction were observed in VO-induced HF model. In this study, we thus investigated the mechanistic effects of increasing mtDNA copy number by the overexpression of TFAM or Twinkle on eccentric hypertrophy and extracellular matrix reconstruction in VO-induced HF in TFAM- and Twinkle-overexpressing mice.

## Materials and Methods

### TG mice and animal model of HF

All procedures and animal care were approved by the Committee on Ethics of Animal Experiment for the Graduate School of Medical and Pharmaceutical Sciences at Kyushu University, and were performed in accordance with the Guidelines for Animal Experiment of Kyushu University (A24–125) and the Guidelines for the Care and Use of Laboratory Animals published by the US National Institutes of Health (NIH, 8^th^ edition, revised in 2011). TG mice overexpressing either human TFAM[12] or murine Twinkle[14,16] under control of β-actin promoter have been described previously. C57BL/6J mice were purchased from Kyudo, Japan. The VO HF model was produced by creating an arteriovenous fistula (AVF) using an established protocol.[17] Briefly, 8- to 10 week-old male mice were anesthetized with a mixture of medetomidine (0.3 mg/kg; Wako Chemicals), midazolam (4 mg/kg; Wako Chemicals), and butorphanol tartrate (5 mg/kg; Wako Chemicals) by intraperitoneal administration according to institutional recommendation. AVFs were created by puncturing the wall separating the aorta and inferior vena cava with a 22-gauge needle. The aorta was clamped superior to the perforation, and the arterial hole was then sealed with cyanoacrylate. AVFs were confirmed by the presence of red oxygenated arterial blood in the vena cava both during the procedure and at sacrifice. Control mice received sham operation without AVF creation. Mice were housed in a temperature- and humidity-controlled room and provided with a commercial diet and water *ad libitum*.

### Echocardiographic and hemodynamic measurements

Under light anesthesia with 1–2% isoflurane, two-dimensional targeted M-mode images were obtained from the short-axis view at the level of papillary muscles by ultrasonography (Vevo2100; VisualSonics). Hemodynamics were measured under anesthesia with tribromoethanol/amylene hydrate (Sigma) by intraperitoneal administration prior to sacrifice, at which point a 1.4 F micromanometer-tipped catheter (Millar Instruments, Inc.) was inserted into the right carotid artery and advanced into the left ventricle (LV) for pressure measurement. After these measurements, mice were euthanized by an overdose of pentobarbital sodium (40 mg/kg; Kyoritsu Seiyaku), and then their hearts and lungs were excised.

### Western blot analyses

Frozen samples were homogenized in RIPA lysis buffer (Thermo Scientific). Mitochondria lysates in RIPA lysis buffer were prepared from the isolated mitochondria derived from heart by centrifugation for western blot analyses of nitrotyrosine and Complex II as an internal control. Equal amounts of protein were then separated on SDS-PAGE and transferred to nitrocellulose membranes. After blocking for 1 h with 5% skim milk in PBS, membranes were incubated with primary antibody at 4°C overnight, followed by incubation with secondary antibody. Primary antibodies used in this study are as follows: Human TFAM (custom made), murine TFAM (sc-23588, Santa Cruz), glutathione peroxidase (ab16798, Abcam), Mn-SOD (sc-18503, Santa Cruz), GAPDH (sc-32233, Santa Cruz), Flag (#2368, Cell Signaling Technology), Complex I (#459100, Invitrogen), Complex II (#459200, Invitrogen), Complex IV (#459600, Invitrogen), nitro-tyrosine (Millipore, AB5411).

### Quantitative real-time polymerase chain reaction analyses

Total DNA and RNA were extracted by standard phenol-chloroform method or RNeasy tissue kit (Qiagen), respectively. Total DNA was treated with MluI (Takara) for mouse mtDNA or BamHI (Takara) for rat mtDNA for 2 h, and the relative amount of mtDNA was quantified by real-time PCR. The antithrombin III (AT III) gene was amplified to estimate the amount of genomic DNA as an internal control. Total RNA was converted to cDNA, and then quantified. PCR mixtures contained 10 ng cDNA and 12 pmol of each primer in a total reaction volume of 30 μL. Ribosomal 18S rRNA was used as an internal control. The primer sequences are shown in S1 Table.

### Measurement of mitochondrial enzymatic activity

Mitochondria were isolated from myocardium using a sucrose gradient and mitochondrial enzymatic activities were measured as described previously.[12] The enzymatic activity of rotenone-sensitive NADH-dehydrogenase (Complex I) was measured by the reduction of decylubiquinone, a ubiquinone analogue. Succinate dehydrogenase (Complex II) enzymatic activity was assessed by the reduction of 2, 6-dichlorophenolindophenol. Coenzyme Q–cytochrome c reductase (Complex III) activity was measured by the reduction of catalyzed cytochrome c catalyzed in the presence of reduced decylubiquinone. Cytochrome c oxidase (Complex IV) activity was assessed by the oxidation of reduced cytochrome c in the presence of dithionite.

### *In situ* zymography

*In situ* zymography was performed on heart tissue samples by using EnzChek Gelatinase/Collagenase Assay Kit (Invitrogen). Briefly, frozen samples in OCT compound were sectioned at 5 μm and air-dried for 30 min. Then, these samples were mounted with PBS or 1.10. phenanthroline monohydrate (10 mmol/L, Sigma p9375) in PBS (used as a negative control) and incubated for 1 h. Next, the samples were washed with PBS, mounted with DQ gelatin (50 μg/mL) and incubated for 3 h in the dark condition. After incubation, the samples were washed and observed under fluorescence microscopy (Olympus IX71; absorption, 495 nm; emission, 515 nm).

### Ex vivo evaluation for ROS using dihydroethidium (DHE)

ROS levels in the heart tissue samples were evaluated ex vivo using a modified version of a previously described method.[5,18] Briefly, frozen tissue samples embedded in OCT compound were sectioned at 10 μm, mounted with 100 μmol/L DHE in PBS and incubated at 37°C for 5 min. Thereafter, the sectioned samples were washed with PBS, fixed with 3% paraformaldehyde at room temperature for 15 min and then washed with PBS again. The washed sectioned samples were then sealed with FluorSave Reagent (Vector Labolatories, H-1000) and observed by fluorescence microscopy (Olympus IX71; absorption, 559 nm, emission, 570 nm).

### Neonatal rat ventricular myocytes isolation and adenovirus vectors harboring human TFAM

Neonatal rat ventricular myocytes were isolated and cultured as previously described.[15] Briefly, neonatal rats were euthanized by an overdose of isoflurane (3–5%), and then hearts were rapidly excised and digested. After digestion of the myocardial tissue with trypsin (Wako) and collagenase type 2 (Worthington), cells were suspended in Dulbecco’s Modified Eagle’s Medium (Sigma-Aldrich) containing 10% fetal bovine serum (FBS, Thermo Scientific), penicillin (Invitrogen) and streptomycin (Invitrogen). Cells were plated twice in 100 mm culture dishes for 70 min each to reduce the number of non-myocytes. Non-adherent cells were plated in culture dishes (Primaria, Falcon). The replication-deficient recombinant adenoviral vector containing hTFAM (AxCAhTFAM) was constructed using the Dual Version Adenovirus Expression Vector Kit from Takara. Adenoviruses were amplified in human embryonic kidney cell line (HEK-293), purified with the Adeno-X Maxi Purification Kit (Clontech), and then titrated with the Adeno-X Rapid Titer kit (Clontech). The viral infection efficiency was >95%, as measured by β-gal staining.

### Complex I-mediated ROS production and resultant gene expression in isolated myocytes

Isolated myocytes were treated with 1 μmol/L rotenone (R8875; Sigma) and incubated for 30 min. After washing with PBS, myocytes were treated with 1 μmol/L DHE, and incubated at 37°C for 10 min. For mRNA analysis, isolated myocytes were treated with 200 nmol/L rotenone for 48 h.

### H9c2 transfection with human TFAM or rat Twinkle-FLAG

The pCMV-hTFAM or pCMV-rTwinkle-FLAG expression vectors were linearized by AseI digestion and transfected into H9c2 rat cardiomyoblasts with Lipofectamine 2000 (Life Technologies). Transfected cells were isolated by G-418 (Nacalai Tesque) selection.

### Complex III-mediated ROS production in H9c2 cell lines

Antimycin A-induced mitoROS production was examined by MitoSOX Red (Molecular Probes) in H9c2 cells as previously described.[19] Briefly, cells were incubated with 5 μmol/L MitoSOX in HBSS containing Ca^2+^/Mg^2+^ for 30 min, washed once, and then treated with 100 μmol/L antimycin A (Sigma, A8674) in HBSS for 30 min.

### In vitro ROS assay using mitochondria isolated from myocardium

Isolated mitochondria (160 μg/mL) were mixed with 50 mmol/L phosphate buffer, 20 mmol/L succinate, 2 μg/mL antimycin A, and 10 μM NBD-Me-TPP, a fluorescent nitroxide switch.[20,21] Fluorescence intensity was measured continuously for 2 min, and the slope defined the rate of free radicals released from mitochondria.

### Statistical Analysis

All data are expressed as mean ± SEM. Multiple groups are compared by one-way ANOVA followed by post-hoc Tukey’s comparison test. Two groups were compared by unpaired Student’s *t*-test.

Additional methods are available in S1 Text.

## Results

### Characterization of TFAM mice and Twinkle mice

**Fig 1:**
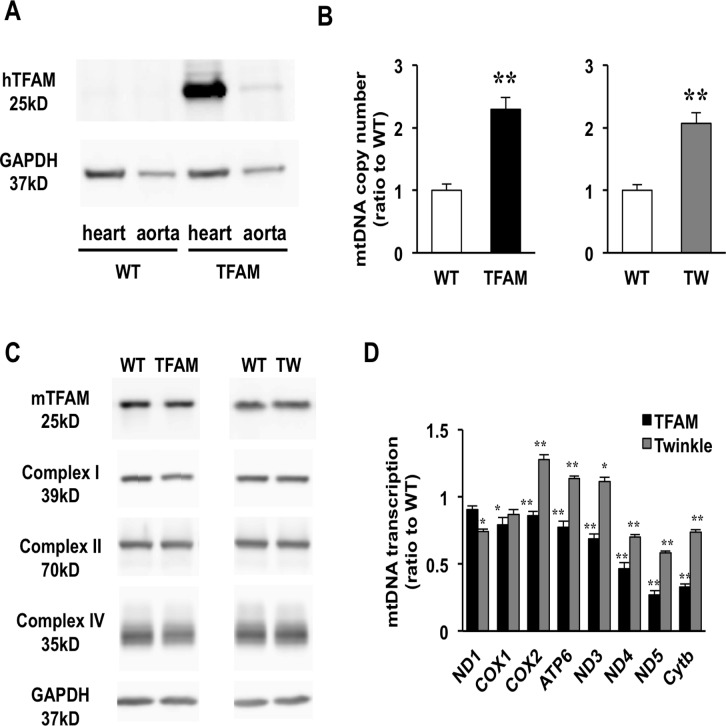
Characterization of TFAM and Twinkle (TW) mice. (A) Expression of human TFAM (hTFAM) in left ventricle (LV) and aorta in TFAM and wild-type (WT) control mice. (B) mtDNA copy number in myocardium from TFAM and TW mice by real-time PCR (n = 4). (C) Expression of endogenous murine TFAM (mTFAM) and mitochondrial complex proteins in LV of TFAM and TW mice. (D) Transcription of mtDNA-encoded genes in TFAM and TW mice (n = 6). Data are expressed as mean ± SEM. *P < 0.05 vs. WT, **P < 0.01 vs. WT, analyzed by Student’s *t-*test.

TG mice overexpressing human TFAM (hTFAM) under control of the β-actin promoter have been described previously.[12] The utilized model VO HF results from an AVF created in the abdominal aorta. As such, we examined hTFAM expression in the aorta of TFAM mice and confirmed a markedly lower expression in the aorta than in the heart (Fig. 1A). Due to the lack of an available Twinkle antibody, we analyzed the transcription of Twinkle in the heart and aorta by real-time PCR, and found an approximately 50-fold increase in the hearts of TG mice as compared to wild-type (WT) mice, without a significant increase in the aorta (S1A Fig.). mtDNA copy number in hearts increased 2.3- and 2.1-fold in TFAM mice and Twinkle mice, respectively, when compared to WT littermates (Fig. 1B); however, no differences in mtDNA copy number were observed in the aortas of TG and WT mice (S1B Fig.). It has been suggested that TFAM expression levels might correlate closely with mtDNA copy number.[22] As such, we compared the expression of endogenous murine TFAM (mTFAM) in the TG mice, and found that endogenous mTFAM was equally expressed in the TG mice compared to WT mice in each strain (Fig. 1C). We further examined the transcription of mtDNA-encoded genes in both TG mouse strains relative to WT mice (Fig. 1D). Notably, mtDNA-encoded gene transcription was suppressed in TFAM mice when compared to WT mice. This result may be explained by two possibilities: (1) hTFAM is unable to mediate the transcription of murine mtDNA-encoded genes, or (2) TFAM overexpression results in the overpackaging of mtDNA and/or D-loop due the increased TFAM/mtDNA ratio.[23] In contrast, Twinkle mice exhibit a variation in mtDNA-encoded gene expression, as some genes were upregulated while others were unchanged or slightly downregulated compared to WT mice. This suggests that mtDNA transcription varies between TFAM and Twinkle mice. Interestingly, despite this disparity, no differences in mitochondrial protein expression were observed (Fig. 1C). In addition, it was previously reported that the ETC enzymatic activities in both TG mice are the same as WT mice.[12,24] These findings demonstrate that neither hTFAM nor Twinkle overexpression bolsters mitochondrial activity at the translational and functional levels, despite the commonality of increased mtDNA copy number in TFAM and Twinkle mice.

### Overexpression of hTFAM or Twinkle equally attenuates eccentric hypertrophy and improves cardiac function under VO

**Fig 2:**
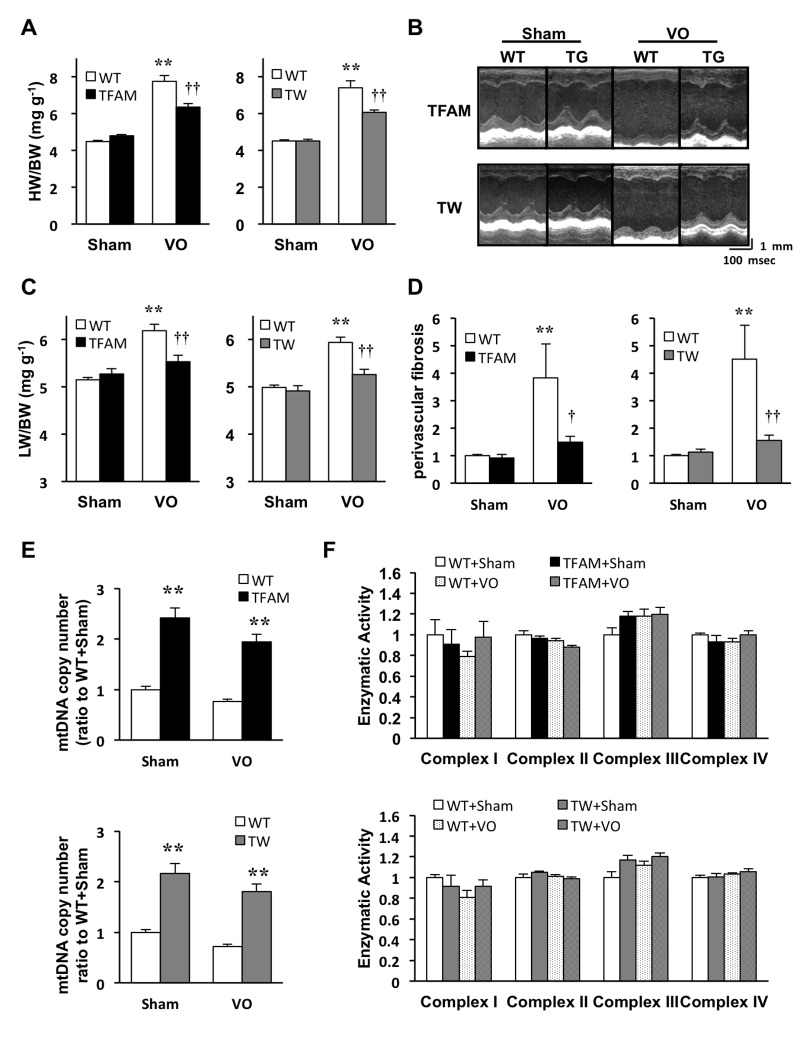
Analysis of TFAM and Twinkle (TW) mice 8 weeks after arteriovenous fistula creation. (A) Heart weight/body weight (HW/BW) (n = 12) (B) M-mode echocardiogram (C) Lung weight/body weight (LW/BW) in TFAM and TW mice (n = 12). (D) Quantification of perivascular fibrosis standardized by vascular circumference (n = 6). (E) mtDNA copy number in left ventricle (LV) of TFAM (upper panel) and TW (lower panel) mice (n = 6). (F) Mitochondrial electron transport chain enzymatic activities in TFAM (upper panel) and TW (lower panel) mice (n = 4–5). Data are expressed as mean ± SEM. *P < 0.05 vs. WT + Sham, **P < 0.01 vs. WT + Sham, ^†^ *P* < 0.01 vs. WT + VO, ^††^ *P* < 0.01 vs. WT + VO, analyzed by one-way ANOVA followed by post hoc Tukey’s test.

We first examined the effects of TFAM- or Twinkle overexpression on LV remodeling at 8 weeks after the AVF procedure. Hemodynamic stress in VO increased the heart mass by approximately 1.6–1.7 fold in WT mice,[25] whereas this observation was attenuated in TFAM and Twinkle mice (Fig. 2A). Echocardiographic images showed severe LV dilatation and dysfunction in WT mice under VO, which were also ameliorated in both TG mice (Fig. 2B, S2 Table). In addition, VO-induced pulmonary congestion, characterized by increased lung mass and left ventricular end-diastolic pressure (LVEDP), was improved in both TG strains compared to WT (Fig. 2C, S3 Table). Histologically, Masson trichrome staining revealed that VO-induced perivascular fibrosis was also suppressed in both TG mice (Fig. 2D, S2A Fig.), although no significant alterations in cross-sectional area were detectable (S2B Fig.). These results validate the major finding that the overexpression of hTFAM or Twinkle attenuates VO-induced eccentric hypertrophy and improves cardiac function to a similar extent with an increase of mtDNA copy number.

### Mitochondrial characteristics in VO-induced myocardial hypertrophy

We previously identified that mtDNA copy number decreased approximately 40% in the failing myocardium of post-MI mice, whereas ETC enzymatic activities are only impaired in WT mice after MI.[12] In this study, mtDNA copy number decreased approximately 25% in the hypertrophied myocardium of WT mice 8 weeks after AVF formation (Fig. 2E), but no significant impairments in ETC enzymatic activities were identified in TG or WT mice (Fig. 2F). These results suggest that preservation of ETC enzymatic activities is not the primary mechanism by which hTFAM or Twinkle overexpression ameliorates eccentric hypertrophy.

### Activation of *in situ* zymography: matrix metalloproteinase (MMP) activity under VO is suppressed in TFAM and Twinkle mice

**Fig 3:**
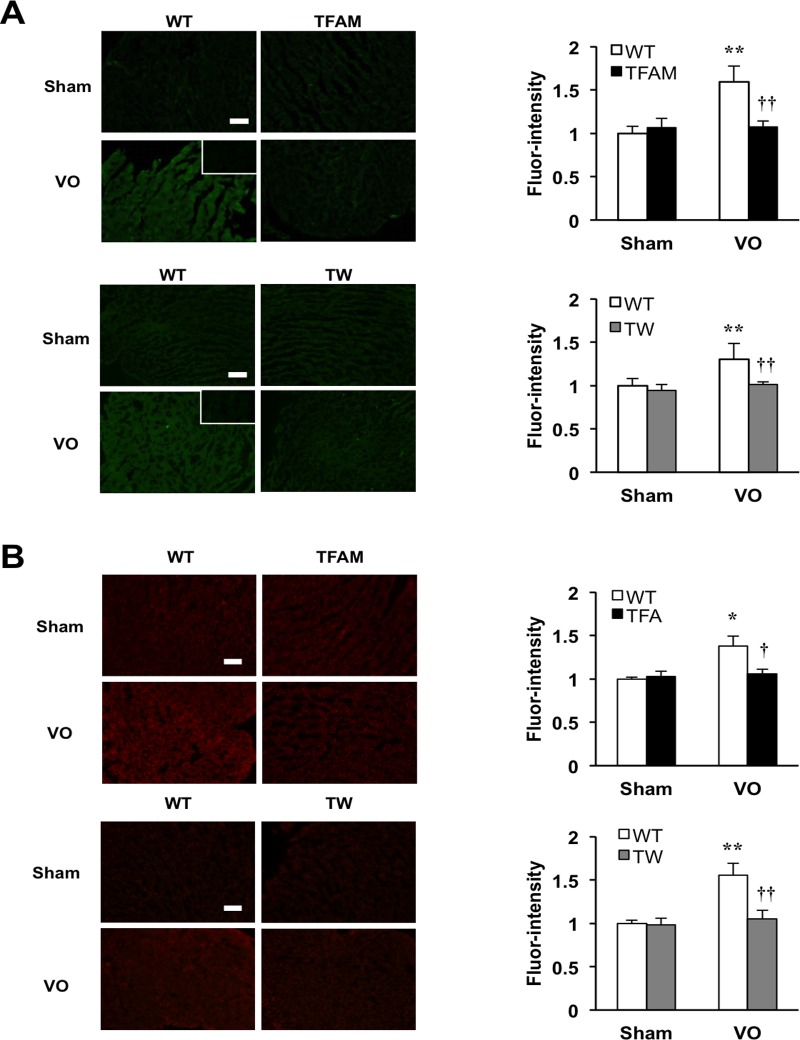
Matrix-metalloproteinase (MMP) gelatinase activity and reactive oxygen species (ROS) production in the heart tissues of TFAM mice and Twinkle (TW) mice at 8weeks after creating arteriovenous fistula. (A) Representative images and quantification of MMP gelatinase activities in LV from TFAM or TW mice at 8 weeks after creating AVF (n = 6), **P < 0.01 vs. WT+Sham, ^†^ *P* < 0.01 vs. WT+VO, ^††^ *P* < 0.01 vs. WT+VO, analyzed by one-way ANOVA followed by post hoc Tukey’s test. (B) Representative images and quantification of Dihydroethidium staining (DHE) staining of LV from TFAM or TW mice (n = 6), **P < 0.01 vs. WT+Sham, ^†^ *P* < 0.01 vs. WT+VO, ^††^ *P* < 0.01 vs. WT+VO, analyzed by one-way ANOVA followed by post hoc Tukey’s test. Scale bar, 100μm. All data are mean±SEM.

Many lines of evidence have established that MMPs harboring gelatinase activity, such as MMP-2 and MMP-9, facilitate the progression of VO-induced HF by degrading extracellular matrix.[26–30] As such, we examined MMP gelatinase activity in left ventricle by *in situ* zymography in both TFAM and Twinkle mice. Notably, basal MMP gelatinase activity was observed in WT mice under VO; however, this was suppressed in TFAM and Twinkle mice (Fig. 3A). Since both MMP-2 and MMP-9 function as redox-sensitive signals at the transcriptional and posttranslational levels,[26] we also examined ROS production in left ventricle using the fluorescence superoxide probe, dihydroethidium (DHE). Significantly, higher levels of ROS were detected in the failing myocardium of WT mice, but not TG mice (Fig. 3B). There results indicate that TFAM and Twinkle TG mice have alterations in VO-induced myocardial MMPs expression and ROS production.

### MMP-2 and MMP-9 mRNA upregulation in acute VO is suppressed in TFAM and Twinkle mice

**Fig 4:**
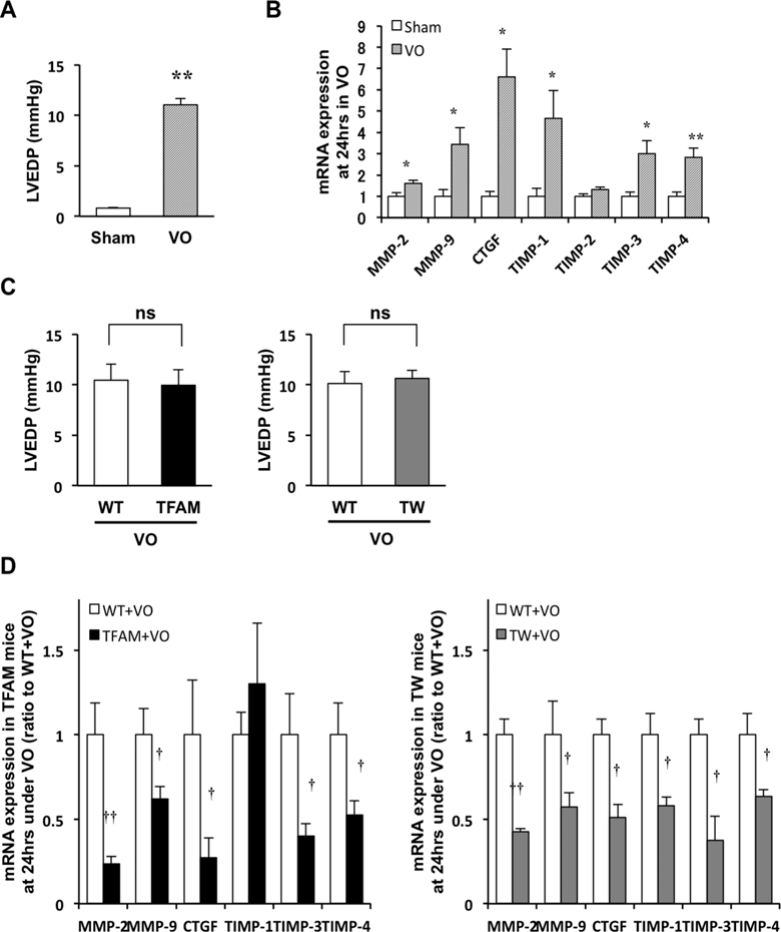
Volume overload (VO)-induced hemodynamic load and mRNA expression of matrix-metalloproteinase (MMP)-2, MMP-9 and other VO-upregulated molecules in TFAM and Twinkle (TW) mice at 24 hours after creating arteriovenous fistula. (A) Left ventricular end diastolic pressure (LVEDP) in C57BL/6J mice after 24 h of volume overload (VO) (n = 4). (B) VO-induced mRNA expression of MMP-2, MMP-9, connective tissue growth factor (CTGF), tissue inhibitor of metalloprotease (TIMP)-1,TIMP-2, TIMP-3, and TIMP-4 in the hearts of C57BL/6J mice, as measured by real-time PCR (n = 4) at 24 h of VO (n = 4). (C) Comparison of LVEDP in WT and transgenic (TG) mice at 24 h after VO creation (n = 6–12). (D) mRNA expression of MMP-2, MMP-9, CTGF, TIMP-1, TIMP-3, and TIMP-4 in the hearts of WT and TG mice under VO, as measured by real-time PCR (n = 6–12). Data are expressed as mean ± SEM. *P < 0.05 vs. Sham, **P < 0.01 vs. Sham, ^†^ *P* < 0.01 vs. WT + VO, ^††^ *P* < 0.01 vs. WT + VO, analyzed by Student’s *t*-test.

The above results support that hTFAM or Twinkle overexpression attenuates eccentric hypertrophy and improves cardiac function under VO independent of mitochondrial ETC enzymatic activities despite of an increase in mtDNA copy number. Thus, we hypothesized that the overexpression of TFAM or Twinkle is sufficient to suppress the molecular signals involved in cardiac remodeling without any associated structural or functional deterioration in the heart and mitochondria. To characterize the gene transcription during the acute phase of VO, we first examined MMP-2 and MMP-9 expression 24 h after AVF creation. LVEDP significantly elevated in mice under VO. (Fig. 4A). Notably, MMP-2 and MMP-9 expression were significantly upregulated after 24 h of VO compared to Sham controls (Fig. 4B). We next examined the effect of increased mtDNA copy number on MMP-2 and MMP-9 upregulation after 24 h of VO. While no differences in LVEDP were observed between WT and TG mice after AVF creation (Fig. 4C), both MMP-2 and MMP-9 were significantly hindered in TG as compared to WT mice under VO (Fig. 4D). Additionally, TFAM or Twinkle overexpression also attenuated the upregulation of other VO-induced genes, including tissue inhibitors of metalloproteinase (TIMP) family members and connective tissue growth factor (CTGF) (Fig. 4B, D).

### TFAM overexpression suppresses mitoROS and the mitoROS-induced upregulation of MMP-2 and MMP-9 in cardiomyocytes

**Fig 5:**
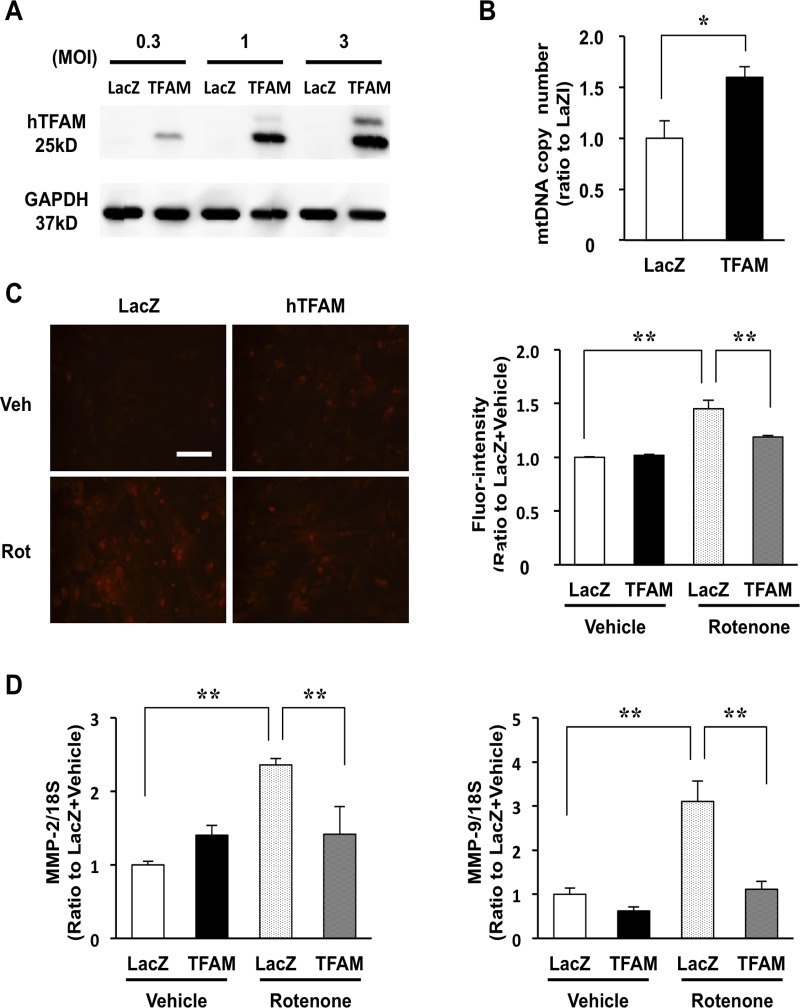
Effect of TFAM overexpression on mitochondrial DNA (mtDNA), mitoROS and mRNA expressions of matrix-metalloproteinase (MMP)-2 and MMP-9 in rat neonatal myocytes. (A) Human TFAM expression at 72 h in neonatal rat ventricular myocytes infected by LacZ (control)-adenovirus or TFAM-adenovirus (multiplicity of infection [MOI] = 0.3, 1, and 3). (B) mtDNA copy number in TFAM-adenovirus infected myocytes at 72 h after infection (1 MOI) (n = 4–5). (C) Rotenone (Rot) or DMSO (Vehicle [Veh])-induced mitoROS detected by dihydroethidium (DHE) staining in LacZ- or TFAM-adenovirus (1 MOI) infected myocytes. Scale bar, 100 μm (left panel). Quantification of fluorescence intensity in LacZ- or TFAM-adenovirus (1MOI) infected myocytes (right panel; n = 3). (D) Rotenone-induced mRNA expressions of matrix-metalloproteinase (MMP)-2 (left panel) and MMP-9 (right panel) in LacZ- or TFAM-adenovirus (1 MOI) infected myocytes (n = 3), as measured by real-time PCR. Data are expressed as mean ± SEM. *P < 0.05, **P < 0.01, analyzed by one-way ANOVA followed by post hoc Tukey’s test.

While our examination revealed that the overexpression of hTFAM or Twinkle suppresses VO-induced gene expression to mitigate HF, the underlying mechanism remains unclear. In the heart, the expression of MMPs are regulated through various signals including endogenous hormones, cytokines, mechanical stretch, and ROS.[26] Mechanical stretch is a main mediator of VO-induced MMP upregulation; however, a significant decrease in MMP expression was observed in both TFAM and Twinkle mice with similar LVEDP. As such, we hypothesized that reduction of mitoROS mainly contributes to the modulation of MMP transcription in both TG mice, based on our previous observation that TFAM overexpression in HeLa cell suppresses mitoROS.[31] To investigate the mechanism underlying the regulation of MMP-2 or MMP-9 expression by mitoROS, and to determine its dependency on TFAM overexpression, we overexpressed human TFAM in isolated rat neonatal cardiomyocytes using an adenoviral vector (Fig. 5A). As expected, hTFAM overexpression increased mtDNA copy number approximately 1.5-fold at 72 h after adenoviral infection (MOI = 1) (Fig. 5B), and inhibited the production of mitoROS following treatment with rotenone, a specific inhibitor of complex I (Fig. 5C). Additionally, rotenone-induced mitoROS elicited the upregulation of MMP-2 and MMP-9 in cardiomyocytes, whereas this effect was suppressed by hTFAM overexpression (Fig. 5D). These results suggest that MMP-2 and MMP-9 are regulated by mitoROS in cardiomyocytes, and that hTFAM overexpression limits the production of mitoROS, suppressing MMP-2 and MMP-9 expression.

### TFAM or Twinkle overexpression suppresses antimycin A-induced mitoROS in H9c2 cells

**Fig 6:**
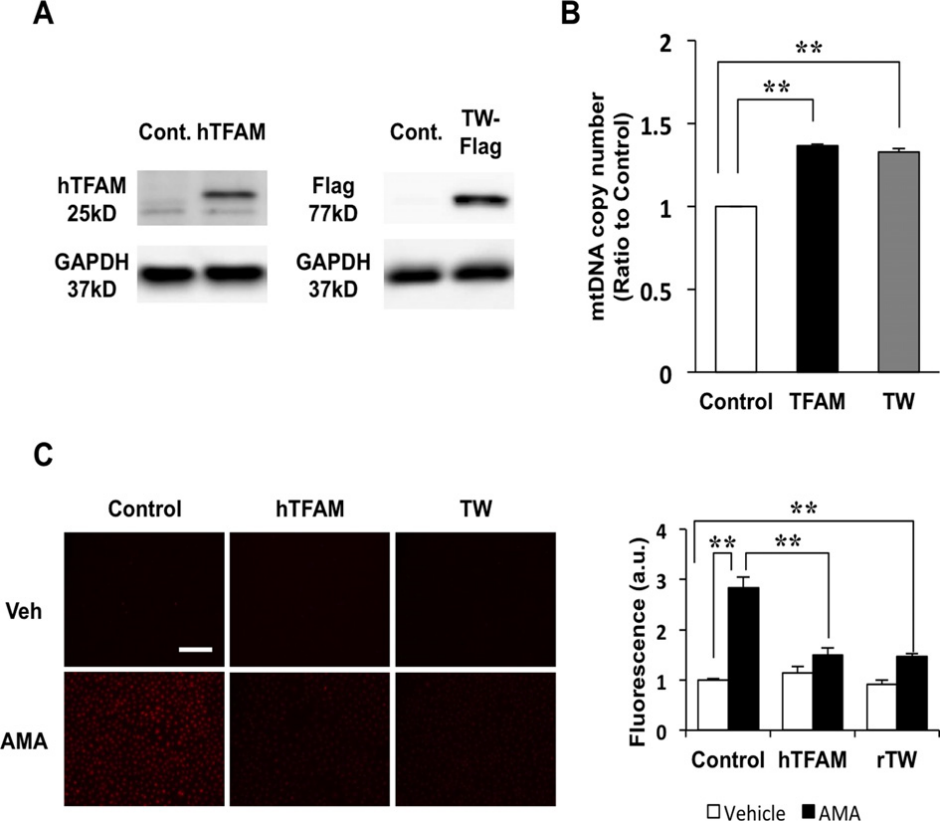
Mitochondrial reactive oxygen species (mitoROS) assay using H9c2 overexpressing human TFAM (hTFAM) or rat Twinkle-Flag (rTwinkle) and mitochondria from TFAM mice and Twinkle (TW) mice. (A) Protein expression of hTFAM in H9c2 overexpressing hTFAM (left panel) and rTwinkle-Flag in H9c2 overexpressing rTwinkle (right panel). (B) Quantification of mtDNA copy number in H9c2 overexpressing hTFAM and rTwinkle by real-time PCR, (C) Antimycin A-induced mitoROS using MitoSOX probe in H9c2 overexpressing hTFAM and rTwinkle, Scale bar, 50 μm (upper panel). Quantification of fluorescence-intensity in the cytoplasm of H9c2 cells overexpressing hTFAM or rTwinkle vs. controls (lower panel; n = 3). Data are expressed as mean ± SEM. *P < 0.05, **P < 0.01, analyzed by one-way ANOVA followed by post hoc Tukey’s test.

To examine the role of mtDNA copy number in the reduction of mitoROS, we generated H9c2 rat cardiomyoblasts that overexpress hTFAM or FLAG-tagged rat Twinkle (rTwinkle). Overexpression of hTFAM or rTwinkle was confirmed by western blot analysis (Fig. 6A), and was accompanied by a significant increase in mtDNA copy number (Fig. 6B). We used these cell lines to investigate the effect of increased mtDNA copy number on the production of mitoROS induced by antimycin A, a specific inhibitor of mitochondrial complex III. As expected, mitoROS were equally suppressed in H9c2 cells overexpressing hTFAM or rTwinkle compared to control cells (Fig. 6C).

### MitoROS and mitochondrial protein oxidation were suppressed in TFAM and Twinkle mice

**Fig 7:**
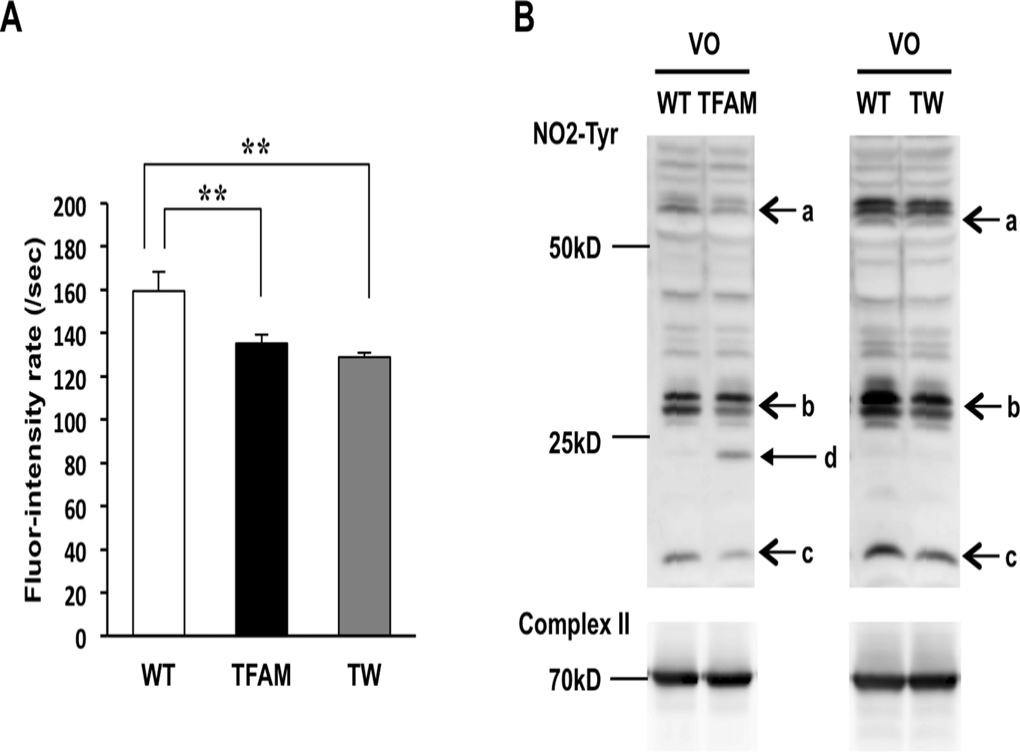
Nitration of mitochondrial protein and oxidized mtDNA extracted from mitochondria of TFAM mice and Twinkle (TW) mice at 8 weeks after creating arteriovenous fistula. (A) Fluorescence intensity rate obtained by an in vitro assay using mitochondria isolated from WT and both transgenic mice together with NBD-Me-TPP (n = 6). Data are expressed as mean ± SEM. *P < 0.05, **P < 0.01, analyzed by one-way ANOVA followed by post hoc Tukey’s test. (B) Western blots of mitochondrial proteins from wild type (WT) and both transgenic (TG) mice using anti-NO_2_-tyrosine antibody and anti-complex II antibody. Arrows a, b, and c identify reduced tyrosine nitration in both TG mice. Arrow d shows an additional nitrated protein (probably nitration of the overexpressed human TFAM protein).

To further examine the effect of increased mtDNA copy number on mitoROS production in vitro, mitochondria isolated from the hearts of TFAM and Twinkle mice were treated with antimycin A in the presence of the fluorescent mitoROS detection reagent, NBD-Me-TPP, which contains triphenyl phosphonium (TPP). NBD-Me-TPP consists of major three sites, a free radical reaction site that acts as a trap for mitoROS (arrow a), fluorescent site (arrow b), and TPP site that facilitates mitochondrial uptake (arrow c) (S3A Fig.).[20] A quencher located between the free radical reaction and fluorescent sites is sequestered upon reacting with mitoROS to yield a fluorescence signal. Continuous fluorescence intensity measurements displayed a linear increase in fluorescence in the presence of antimycin A (S3B Fig.), and the slope of the fluorescence intensity time curve was dependent on the amount of mitochondria-derived free radicals. In accordance with the previous findings, the amount of free radicals released in mitochondria isolated from TFAM and Twinkle mice were markedly lower compared to that of WT controls (Fig. 7A). Nitrotyrosine (NO2-Tyr) is an oxidative modification resulting from nitration of tyrosine that is induced by reactive nitrogen species, and can be used as an indicator of redox in mitochondria. As such, we examined the presence of NO2-Tyr in proteins extracts from mitochondria isolated from the hearts of TFAM and Twinkle mice, and found an overall reduction of NO2-Tyr residues in TG mice compared to WT controls (Fig. 7B, arrows a, b, or c). These results demonstrate that hTFAM or Twinkle overexpression is sufficient to limit not only mitoROS but also the oxidation of mitochondrial proteins.

## Discussion

Mitochondrial DNA is organized as a circular, closed, double-stranded DNA and encodes 13 ETC subunit proteins, 22 transfer RNAs, and two ribosomal RNAs. One mitochondrion has multiple mtDNA copies, the depletion of which is associated with various diseases.[32] Other research groups as well as we have shown that mtDNA depletion is associated with HF in both humans and experimental animal models.[4,5,33,34] Using two TG mouse strains that possess increased mtDNA copy numbers through different mechanisms, this study demonstrates that TFAM or Twinkle overexpression increases mtDNA copy number, attenuates VO-induced eccentric hypertrophy and improves cardiac function, and also suppresses the production of mitoROS and the subsequent redox-sensitive signals.

Recent studies have established the role of ROS and redox-sensitive signals in LV dilatation during cardiac remodeling, which is accompanied by cellular alignment, myocyte elongation, and reconstruction.[35] While mitoROS have attracted a great deal of attention in therapeutic strategy for heart failure,[36] little is known about their effects on cardiac remodeling. In this study, we demonstrate that MMP-2 and MMP-9—both of which have predominant roles in the progression of VO-induced HF—were regulated by mitoROS in myocytes, and that the overexpression of either TFAM or Twinkle suppressed their expression by limiting mitoROS. In our current pursuit towards the clinical application of rhTFAM protein in heart failure, we have succeeded in increasing mtDNA copy number in vivo with the administration of human recombinant TFAM protein (rhTFAM) (S4 Fig.), emphasizing the feasibility of rhTFAM therapy in the future.

Despite these analyses, the mechanism of how the overexpression of TFAM or Twinkle limits mitoROS remains to be elucidated. We examined the presence of major mitoROS scavenging enzymes, such as manganese superoxide dismutase (Mn-SOD), glutathione peroxidase (GPx), and peroxiredoxin (Prx); however, no differences were observed at the gene or protein level (S5A, B Fig.). Since the overexpression of either TFAM or Twinkle was sufficient to limit mitoROS levels, we initially expected to see a decrease in oxidized mtDNA in both TG mice, yet oxidized mtDNA in myocardium was increased in both TG mice after 8 weeks of VO, as measured by real-time PCR with using 8-oxoguanine glycosylase (OGG1) (S6A Fig.). In addition, immunohistochemistry using an 8-hydroxy-2'-deoxyguanosine (8-OHdG) antibody to detect oxidized mtDNA revealed increased cytoplasmic staining in both TG mice compared to WT mice (S6B Fig.). Pohjoismaki et al. recently reported that the presence of 8-oxoG is directly proportional to the amount of mtDNA in both Twinkle and WT mice.[37] Indeed, 8-oxoG per mitochondrial protein increased in accordance with increased mtDNA copy number compared to WT controls, as measured by Southwestern blotting (S6C Fig.). Therefore, we speculate that mtDNA limits mitoROS by acting as a preferential substrate for ROS-mediated oxidation, similar to the functions of mitoQ (S6D Fig.). Nevertheless, further investigation is necessary to conclusively determine the significance of oxidized mtDNA in both TG mice. Furthermore, Stuart et al. demonstrated that OGG-1 null mice exhibit more oxidized mtDNA without any deterioration of mitochondrial function and cardiac function, though oxidized mtDNA is believed as harmful.[38] Our results from both TG mice also show that elevations of oxidized mtDNA are not always accompanied with deterioration of mitochondria function and cardiac function.

In addition, a molecular foundation for the excessive mitoROS production observed under VO remains to be elucidated. Mitochondria-localized NADPH oxidase 4 (Nox4) was increased significantly after 14 days but unaltered at 1 day of VO, which suggests Nox 4 might be a mediator of excessive mitoROS production in myocardium during chronic VO but not in acute phase of VO (S7 Fig.).[5] While we speculate that major cause of excessive mitoROS production is attributed to increased oxygen consumption in acute phase of VO, Gladden et al. previously demonstrated that mitochondrial dysfunction, which is characterized by a reduction of State 3 mitochondrial respiration, occurs 24 h after VO initiation.[39] Although no impairments were observed in the enzymatic activities of ETC complexes after 24 h of VO in our measurements (data not shown), potential alterations in mitochondrial function might cause excessive mitoROS to elicit redox-sensitive signals during the acute phase of VO.

## Conclusions

TFAM or Twinkle overexpression increases mtDNA copy number and facilitates cardioprotection associated with limited mitochondrial oxidative stress. Given that mtDNA quantity decreases in both HF patients and animal models, and mitoROS is one of the prospective therapeutic targets for heart failure, our findings will provide new insights into the pathophysiology in heart failure, and propose a potential therapeutic strategy targeting mtDNA and mitoROS for heart failure.

## Supporting Information

S1 Fig mRNA expression of Twinkle in Twinkle-transgenic mice and mtDNA copy number in aorta from hTFAM-transgenic mice and Twinkle-transgenic mice. (A) mRNA expression of Twinkle in heart and aorta from Twinkle-transgenic mice compared to wild type (WT) mice (n = 3), *P < 0.05 vs. heart from WT, **P < 0.01 vs. heart from WT, analyzed by one-way ANOVA followed by post hoc Tukey’s test. (B) Quantification of mtDNA copy number in aorta from hTFAM-transgenic mice and Twinkle-transgenic mice by real-time PCR (n = 4–11), analyzed by Student’s t-test. ns; not significant.
(PDF)

S2 Fig Perivascular fibrosis and cross-sectional area of myocytes in LV eight weeks after AVF creation. (A) Representative images of Masson-Trichrome staining of myocardium sections demonstrating perivascular fibrosis in TFAM and TW mice. Scale bar, 100 μm. (B) Cross-sectional area of myocytes in LV of TFAM or TW mice at 8 weeks after creating AVF measured on hematoxylin-eosin (HE) stained sections (n = 6), analyzed by one-way ANOVA followed by post hoc Tukey’s test. All data are mean±SEM.
(PDF)

S3 Fig NBD-TPP-Me and in vitro ROS assay using isolated mitochondria. (A) Structure of TPP-NBD-Me. (B) Representative raw data of fluor-intensity measurements with mitochondria derived from WT, TFAM, and Twinkle mice.
(PDF)

S4 Fig hTFAM administration to mice. mtDNA copy number in heart of recombinant human TFAM (rhTFAM) administrated mice compared to controls (n = 3), measured by real-time PCR, *P < 0.05, ** *P* < 0.01 vs. control, analyzed by one-way ANOVA followed by post hoc Tukey’s test.
(PDF)

S5 Fig Expression of mitochondrial scavenging enzymes in hTFAM-transgenic mice (TFAM mice) and Twinkle-transgenic mice (TW mice). (A) Western blots of Mn-SOD and GPx-1 in TFAM and TW mice. (B) Gene expressions associated with redox regulation in TFAM and TW mice (n = 6), *P < 0.05, ** *P*<0.01 vs. WT, analyzed by Student’s t-test.
(PDF)

S6 Fig Oxidized mtDNA extracted from mitochondria of TFAM mice and Twinkle (TW) mice at 8 weeks after creating arteriovenous fistula. (A) Oxidized mtDNA copy number in the left ventricle of WT and both TG mice at 8 weeks by real-time PCR method using OGG-1 (n = 5). (B) Representative images of immunohistochemistry using 8-oxo-2'-deoxyguanosine (8 OH-dG) antibody on LV of WT and both TG mice at 8 weeks. (C) Southwestern blots of mtDNA using 8-oxo-guanine antibody (upper panel), Total amount of mtDNA using ethidium bromide (EtBr) in mitochondrial lysates obtained from left ventricle tissues of equal mass from WT and both TG mice (middle panel), Coomassie Brilliant Blue stain as a control (lower panel). (D) Scheme of the potential mechanism underlying mtDNA-dependent ROS reduction. Data are expressed as mean ± SEM. *P < 0.05 vs. WT + VO, analyzed by Student’s *t*-test.
(PDF)

S7 Fig NADPH oxidase 4 (Nox4) expression in volume overload. mRNA expression of NADPH oxidase 4 (Nox4) in volume overload (n = 4), by real-time PCR, *P < 0.05, ** *P* < 0.01 vs. 0 day, one-way ANOVA followed by post hoc Tukey’s test. ns, not significant. All data are mean ± SEM.
(PDF)

S1 Table List of primer sequences used in this study. Cytb, cytochrome b; COX I, cyclooxygenase I; AT III, antithrombin III; MMP, matrix metalloproteinase; TIMP, tissue inhibitor of metalloproteinase; CTGF, connective tissue growth factor; GPx, glutathione peroxidase; Mn-SOD, manganese superoxide dismutase; Prx, peroxiredoxin; Nox4, NADPH oxidase 4.
(DOCX)

S2 Table Hemodynamic measurement at 8 weeks after creating arteriovenous fistula (AVF). TFAM, human mitochondrial transcription factor A-transgenic mice; TW, Twinkle-transgenic mice; WT, wild type mice; VO, volume overload; Sham, sham-operated; HR, heart rate; mAoP, mean aortic pressure; LVEDP, left ventricular end diastolic pressure. Data are expressed as mean ± SEM. *P < 0.05 vs. WT+Sham, **P < 0.01 vs. WT+Sham, ^†^ *P* < 0.01 vs. WT+VO, ^††^ *P* < 0.01 vs. WT+VO, analyzed by one-way ANOVA followed by post hoc Tukey’s test.
(DOCX)

S3 Table Echocardiographic measurement at 8 weeks after creating arteriovenous fistula (AVF). LVDd, left ventricular diameter diastole; LVDs, left ventricular diameter systole; LVEF, left ventricular ejection fraction; IVS, interventricular septum; LVPW, left ventricular posterior wall; TFAM, human mitochondrial transcription factor A-transgenic mice; TW, Twinkle-transgenic mice; WT, wild type mice; VO: volume overload; Sham, sham-operated. Data are expressed as mean ± SEM. *P < 0.05 vs. WT+Sham, **P < 0.01 vs. WT+Sham, ^†^ *P* < 0.01 vs. WT+VO, ^††^ *P* < 0.01 vs. WT+VO, analyzed by one-way ANOVA followed by post hoc Tukey’s test.
(DOCX)

S1 Text Additional methods. (DOCX)
