# Supplementary material for: Overexpression of TFAM or Twinkle Increases mtDNA Copy Number and Facilitates Cardioprotection Associated with Limited Mitochondrial Oxidative Stress
Source: PLoS One. 2015 Mar 30;10(3):e0119687. doi: 10.1371/journal.pone.0119687 (PMC4379048; doi:10.1371/journal.pone.0119687)

S1 Fig. **mRNA expression of Twinkle in Twinkle-transgenic mice and mtDNA copy number in aorta from hTFAM-transgenic mice and Twinkle-transgenic mice.**

A

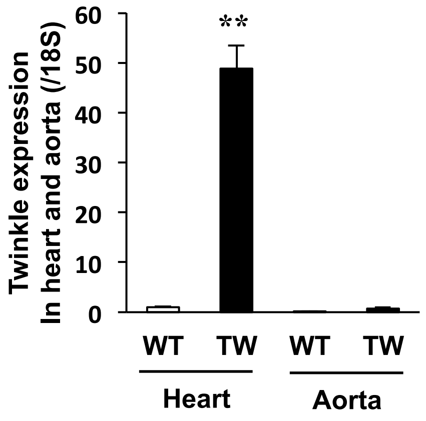

B

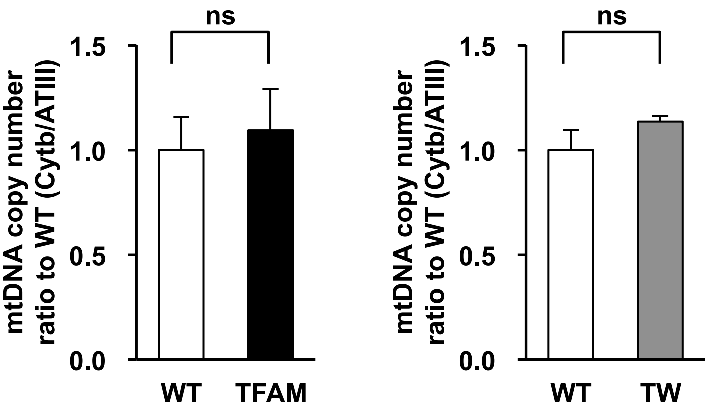

Supplement: S1 Fig — (A) mRNA expression of Twinkle in heart and aorta from Twinkle-transgenic mice compared to wild type (WT) mice (n = 3), *P < 0.05 vs. heart from WT, **P < 0.01 vs. heart from WT, analyzed by one-way ANOVA followed by post hoc Tukey’s test. (B) Quantification of mtDNA copy number in aorta from hTFAM-transgenic mice and Twinkle-transgenic mice by real-time PCR (n = 4–11), analyzed by Student’s t-test. ns; not significant. (PDF) [file pone.0119687.s001.pdf]
