# Supplementary material for: Overexpression of TFAM or Twinkle Increases mtDNA Copy Number and Facilitates Cardioprotection Associated with Limited Mitochondrial Oxidative Stress
Source: PLoS One. 2015 Mar 30;10(3):e0119687. doi: 10.1371/journal.pone.0119687 (PMC4379048; doi:10.1371/journal.pone.0119687)

# S2 Fig. Perivascular fibrosis and cross-sectional area of myocytes in LV eight weeks after AVF creation

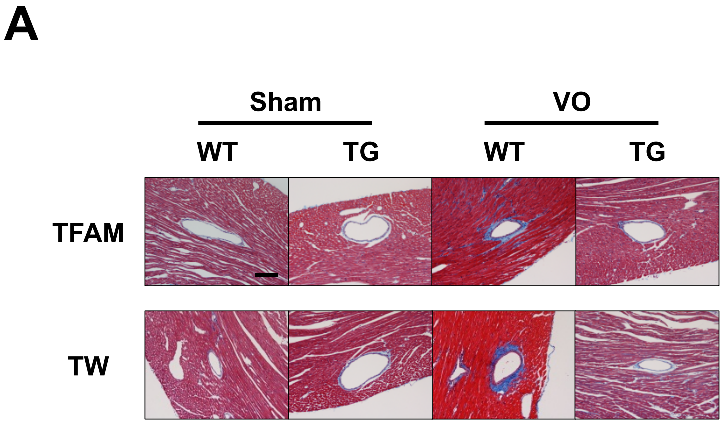


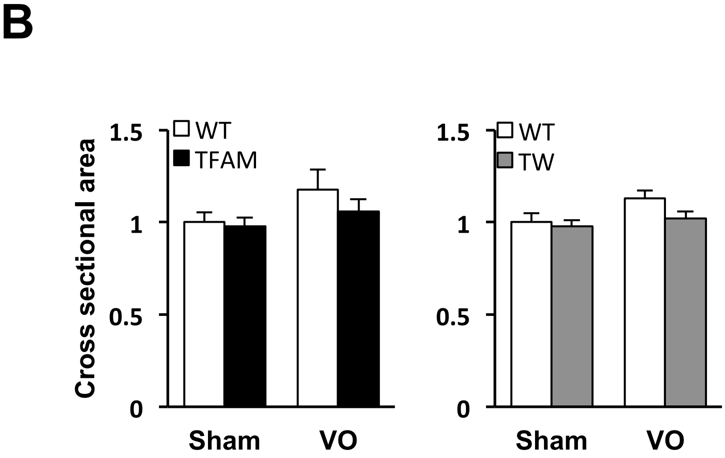

Supplement: S2 Fig — (A) Representative images of Masson-Trichrome staining of myocardium sections demonstrating perivascular fibrosis in TFAM and TW mice. Scale bar, 100 μm. (B) Cross-sectional area of myocytes in LV of TFAM or TW mice at 8 weeks after creating AVF measured on hematoxylin-eosin (HE) stained sections (n = 6), analyzed by one-way ANOVA followed by post hoc Tukey’s test. All data are mean±SEM. (PDF) [file pone.0119687.s002.pdf]
