# Supplementary figures and images for: Overexpression of TFAM or Twinkle Increases mtDNA Copy Number and Facilitates Cardioprotection Associated with Limited Mitochondrial Oxidative Stress
Source: PLoS One. 2015 Mar 30;10(3):e0119687. doi: 10.1371/journal.pone.0119687 (PMC4379048; doi:10.1371/journal.pone.0119687)

S3 Fig. NBD-TPP-Me and in vitro ROS assay using isolated mitochondria

A

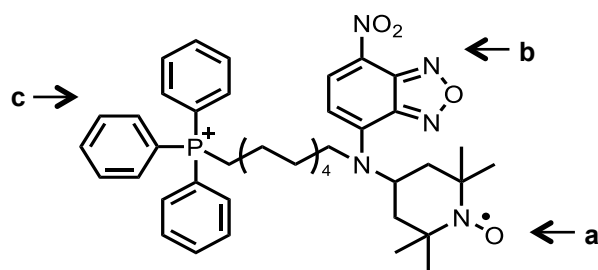

B

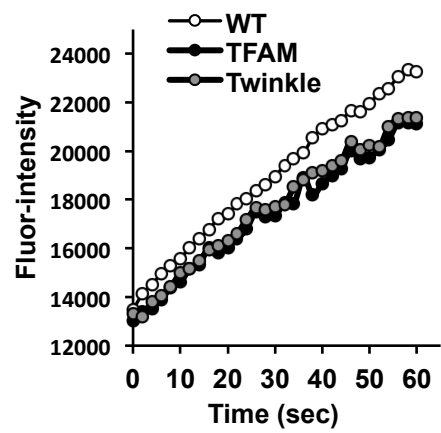

Supplement: S3 Fig — (A) Structure of TPP-NBD-Me. (B) Representative raw data of fluor-intensity measurements with mitochondria derived from WT, TFAM, and Twinkle mice. (PDF) [file pone.0119687.s003.pdf]

S4 Fig. hTFAM administration to mice

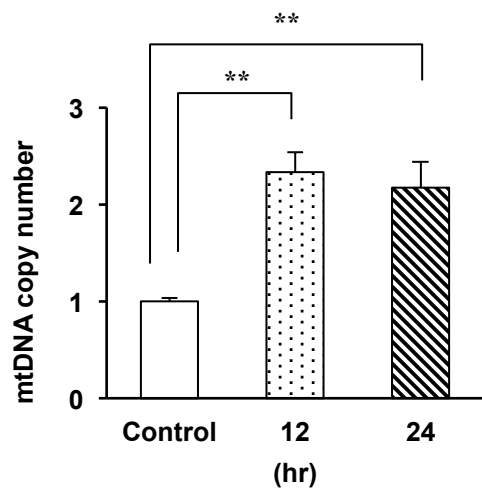

Supplement: S4 Fig — mtDNA copy number in heart of recombinant human TFAM (rhTFAM) administrated mice compared to controls (n = 3), measured by real-time PCR, *P < 0.05, ** P < 0.01 vs. control, analyzed by one-way ANOVA followed by post hoc Tukey’s test. (PDF) [file pone.0119687.s004.pdf]

**S7 Fig. NADPH oxidase 4 (Nox4) expression in volume overload**

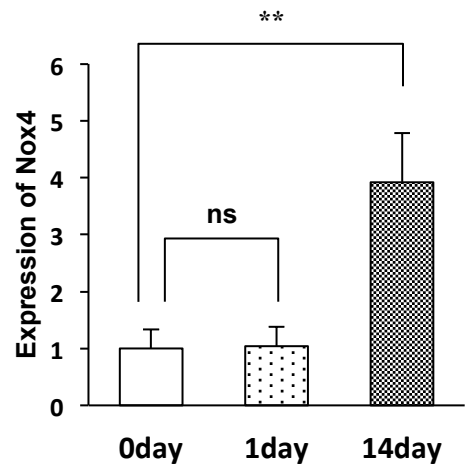

Supplement: S7 Fig — mRNA expression of NADPH oxidase 4 (Nox4) in volume overload (n = 4), by real-time PCR, *P < 0.05, ** P < 0.01 vs. 0 day, one-way ANOVA followed by post hoc Tukey’s test. ns, not significant. All data are mean ± SEM. (PDF) [file pone.0119687.s007.pdf]
