# Supplementary material for: Overexpression of TFAM or Twinkle Increases mtDNA Copy Number and Facilitates Cardioprotection Associated with Limited Mitochondrial Oxidative Stress
Source: PLoS One. 2015 Mar 30;10(3):e0119687. doi: 10.1371/journal.pone.0119687 (PMC4379048; doi:10.1371/journal.pone.0119687)

S5 Fig. Expression of mitochondrial scavenging enzymes in hTFAM-transgenic mice (TFAM mice) and Twinkle-transgenic mice (TW mice)

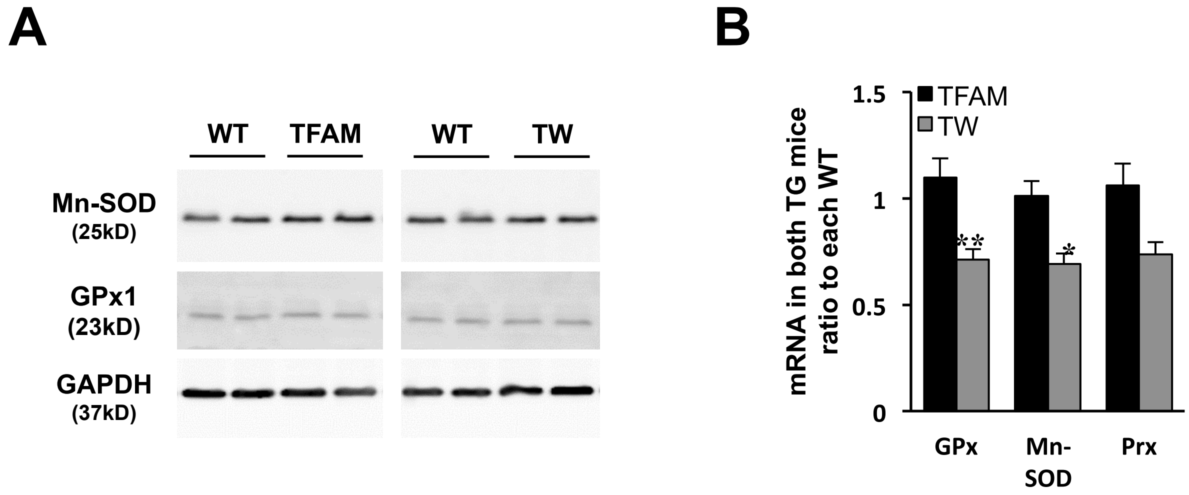

Supplement: S5 Fig — (A) Western blots of Mn-SOD and GPx-1 in TFAM and TW mice. (B) Gene expressions associated with redox regulation in TFAM and TW mice (n = 6), *P < 0.05, ** P<0.01 vs. WT, analyzed by Student’s t-test. (PDF) [file pone.0119687.s005.pdf]
