# Supplementary material for: Overexpression of TFAM or Twinkle Increases mtDNA Copy Number and Facilitates Cardioprotection Associated with Limited Mitochondrial Oxidative Stress
Source: PLoS One. 2015 Mar 30;10(3):e0119687. doi: 10.1371/journal.pone.0119687 (PMC4379048; doi:10.1371/journal.pone.0119687)

S6 Fig. Oxidized mtDNA extracted from mitochondria of TFAM mice and Twinkle (TW) mice at 8 weeks after creating arteriovenous fistula

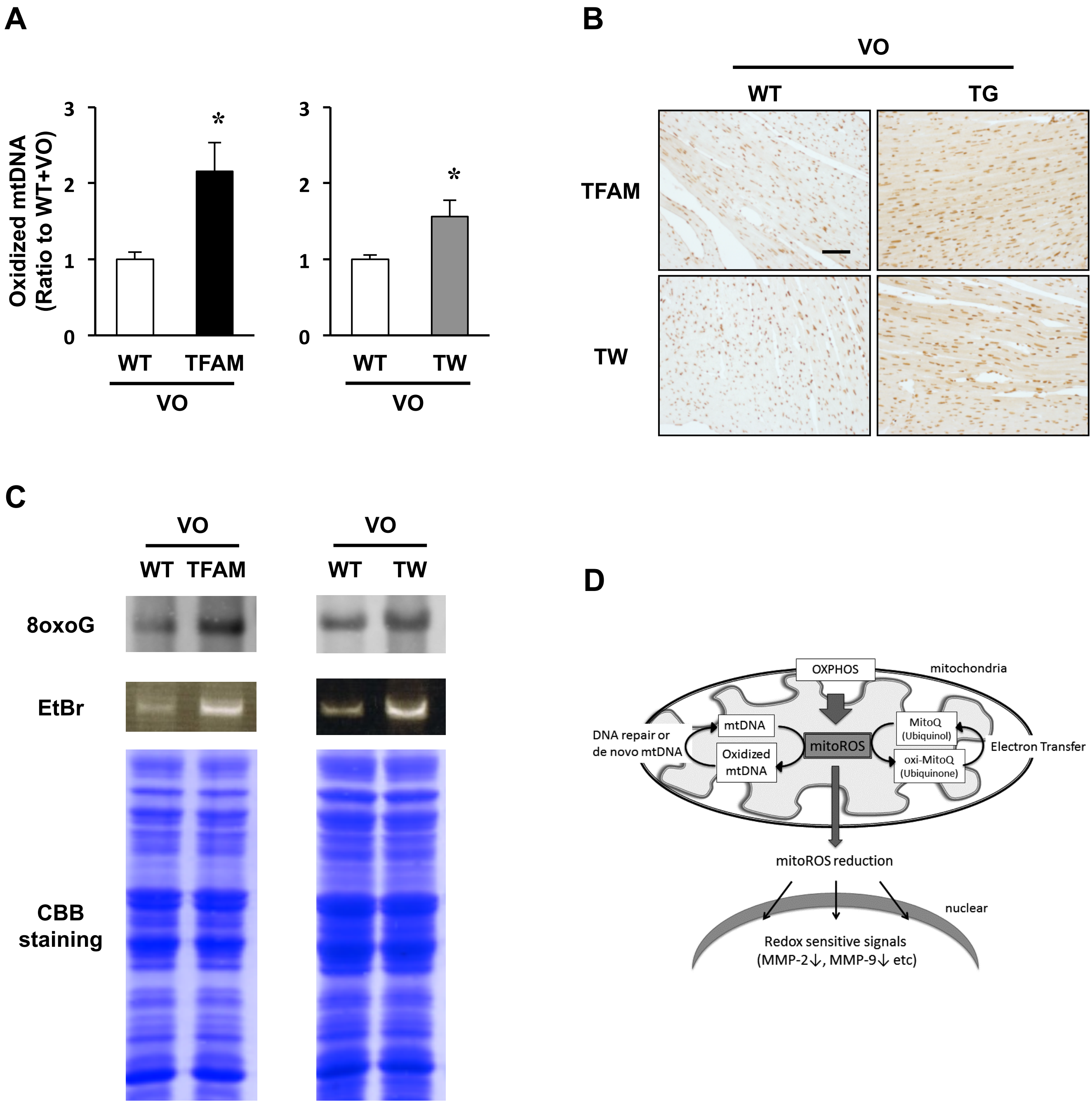

Supplement: S6 Fig — (A) Oxidized mtDNA copy number in the left ventricle of WT and both TG mice at 8 weeks by real-time PCR method using OGG-1 (n = 5). (B) Representative images of immunohistochemistry using 8-oxo-2'-deoxyguanosine (8 OH-dG) antibody on LV of WT and both TG mice at 8 weeks. (C) Southwestern blots of mtDNA using 8-oxo-guanine antibody (upper panel), Total amount of mtDNA using ethidium bromide (EtBr) in mitochondrial lysates obtained from left ventricle tissues of equal mass from WT and both TG mice (middle panel), Coomassie Brilliant Blue stain as a control (lower panel). (D) Scheme of the potential mechanism underlying mtDNA-dependent ROS reduction. Data are expressed as mean ± SEM. *P < 0.05 vs. WT + VO, analyzed by Student’s t-test. (PDF) [file pone.0119687.s006.pdf]
