# Supplementary material for: Overexpression of TFAM or Twinkle Increases mtDNA Copy Number and Facilitates Cardioprotection Associated with Limited Mitochondrial Oxidative Stress
Source: PLoS One. 2015 Mar 30;10(3):e0119687. doi: 10.1371/journal.pone.0119687 (PMC4379048; doi:10.1371/journal.pone.0119687)
Supplement: S1 Table — Cytb, cytochrome b; COX I, cyclooxygenase I; AT III, antithrombin III; MMP, matrix metalloproteinase; TIMP, tissue inhibitor of metalloproteinase; CTGF, connective tissue growth factor; GPx, glutathione peroxidase; Mn-SOD, manganese superoxide dismutase; Prx, peroxiredoxin; Nox4, NADPH oxidase 4. (DOCX) [file pone.0119687.s008.docx]

**Supporting Information S1 Table**

**Table S1. List of primer sequences used in this study.**

Cytb, cytochrome b; COX I, cyclooxygenase I; AT III, antithrombin III; MMP, matrix metalloproteinase; TIMP, tissue inhibitor of metalloproteinase; CTGF, connective tissue growth factor; GPx, glutathione peroxidase; Mn-SOD, manganese superoxide dismutase; Prx, peroxiredoxin; Nox4, NADPH oxidase 4
