# Supplementary material for: Overexpression of TFAM or Twinkle Increases mtDNA Copy Number and Facilitates Cardioprotection Associated with Limited Mitochondrial Oxidative Stress
Source: PLoS One. 2015 Mar 30;10(3):e0119687. doi: 10.1371/journal.pone.0119687 (PMC4379048; doi:10.1371/journal.pone.0119687)
Supplement: S3 Table — LVDd, left ventricular diameter diastole; LVDs, left ventricular diameter systole; LVEF, left ventricular ejection fraction; IVS, interventricular septum; LVPW, left ventricular posterior wall; TFAM, human mitochondrial transcription factor A-transgenic mice; TW, Twinkle-transgenic mice; WT, wild type mice; VO: volume overload; Sham, sham-operated. Data are expressed as mean ± SEM. *P < 0.05 vs. WT+Sham, **P < 0.01 vs. WT+Sham, † P < 0.01 vs. WT+VO, †† P < 0.01 vs. WT+VO, analyzed by one-way ANOVA followed by post hoc Tukey’s test. (DOCX) [file pone.0119687.s010.docx]

**Supporting Information S3 Table**

**S3 Table. Echocardiographic measurement at 8 weeks after creating arteriovenous fistula (AVF).**

LVDd, left ventricular diameter diastole; LVDs, left ventricular diameter systole; LVEF, left ventricular ejection fraction; IVS, interventricular septum; LVPW, left ventricular posterior wall; TFAM, human mitochondrial transcription factor A-transgenic mice; TW, Twinkle-transgenic mice; WT, wild type mice; VO: volume overload; Sham, sham-operated. Data are expressed as mean ± SEM. ^*^*P* <0.05 vs. WT+Sham, ^**^*P* <0.01 vs. WT+Sham, ^†^*P* <0.01 vs. WT+VO, ^††^*P* <0.01 vs. WT+VO, analyzed by one-way ANOVA followed by post hoc Tukey’s test.
