# Supplementary material for: Overexpression of TFAM or Twinkle Increases mtDNA Copy Number and Facilitates Cardioprotection Associated with Limited Mitochondrial Oxidative Stress
Source: PLoS One. 2015 Mar 30;10(3):e0119687. doi: 10.1371/journal.pone.0119687 (PMC4379048; doi:10.1371/journal.pone.0119687)
Supplement: S1 Text — (DOCX) [file pone.0119687.s011.docx]

**S1. Additional methods**

**Immunohistochemistry of 8-hydroxy-2’-deoxyguanosine (8-OHdG) in mtDNA**

Immunohistochemistry was performed as described previously.[[1](#_ENREF_1)] After deparaffinization, tissue sections were autoclaved for antigen retrieval, incubated with RNase at 37°C for 1 h, and then blocked with 1% Block Ace (DS Pharma Biomedical) in H_2_O for 30 min. Anti-8-OHdG antibody (Japanese Institute for the Control of Aging) was diluted in PBS (1:100) and applied to the sections overnight at 4°C. After endogenous peroxidase deactivation by 0.3% H_2_O_2_ for 15 min, biotinylated secondary antibody (anti-mouse IgG; BD Pharmingen) was applied for 1 h, followed by streptavidin-HRP (BD Pharmingen) for 30 min at room temperature.

**Oxidized mtDNA copy number**

Oxidized mtDNA copy number was measured with 8-oxoguanine DNA glycosylase (OGG1) as described previously with some modifications.[[2](#_ENREF_2)] Total DNA was treated with OGG1 (New England Biolabs) for 1 h after MluI treatment. Cycle threshold (Ct) values of the OGG1-treated sample and untreated control were measured by real-time PCR. Oxidized mtDNA copy number in each sample was calculated from the difference of Ct (ΔCt) between the OGG1-treated and untreated samples in accordance with the calculation formula as below.

Oxidized mtDNA copy number was calculated as follow.

First, we defines variables (*Y, Y’* and *ΔY*) as below.

*Y* = mtDNA copy number

*Y’* = mtDNA copy number after OGG1 treatment

(non-oxidized mtDNA copy number)

*ΔY* = Oxidized mtDNA copy number

Then, *ΔY=Y-Y’*

If we determines detection threshold (T) in real-time PCR analysis,

*Y*=$\frac{\alpha T}{2^{CT}}$ , *Y*’’=$\frac{\alpha T}{2^{CT(OGG)}}$

(α: constant, CT: CT value, CT(OGG); OGG-treated CT value)

*ΔY*=$\alpha T\left( \frac{1}{2^{CT}}-\frac{1}{2^{CT(OGG)}} \right)$

=$\alpha T\left( \frac{2^{CT(OGG)}-2^{CT}}{2^{CT+CT(OGG)}} \right)$

Oxidized mtDNA copy number in TG+VO (=*ΔY*(TG+VO)/*ΔY*(WT+VO))

=$\left( \frac{2^{CT(OGG, TG+VO)}-2^{CT\left( TG+VO \right)}}{2^{CT(TG+VO)+CT(OGG, TG+VO)}} \right)$ / $\left( \frac{2^{CT(OGG WT+VO)}-2^{CT(WT+VO)}}{2^{CT(WT+VO)+CT(OGG, WT+VO)}} \right)$

**Oxidized mtDNA Southwestern analysis**

Oxidized mtDNA was analysed by Southwestern blotting as previously described.[[3](#_ENREF_3)] Mitochondria were isolated from fresh LV samples (10 mg) by centrifugation, and mtDNA was then purified using the mtDNA Extractor CT Kit (Wako Chemicals) and suspended in 20 μL nuclease-free water. mtDNA in 10 μL suspension was digested with MluI (Takara), separated by 0.7% agarose gel, and then transferred to Hybond-N+ membranes (Amersham) overnight. Membranes were blocked with 5% skim milk in PBS, incubated overnight with anti-8-oxoguanine antibody (ab64548, Abcam), and then probed with an anti-mouse secondary antibody. CBB stains were achieved using mitochondria lysates from the LV tissue of equal mass.

**Administration of recombinant human TFAM (rhTFAM) for mice**

rhTFAM was prepared as previously described.[[4](#_ENREF_4)] rhTFAM in saline (3μg/gBW) was administrated for mice via tail vein. Mice were sacrificed at 12 hours or 24 hours after single administration of rhTFAM, and their hearts were excised. Control mice were sacrificed after administration of vehicle only (saline).

**References**

1. Stuart JA (2009) Mitochondrial DNA : Methods and Protocols. Catharines, Ontario, Canada: Humana Press. 199-212 p.

2. Lin CS, Wang LS, Tsai CM, Wei YH (2008) Low copy number and low oxidative damage of mitochondrial DNA are associated with tumor progression in lung cancer tissues after neoadjuvant chemotherapy. Interact Cardiovasc Thorac Surg 7: 954-958.

3. Pohjoismaki JL, Williams SL, Boettger T, Goffart S, Kim J, et al. (2013) Overexpression of Twinkle-helicase protects cardiomyocytes from genotoxic stress caused by reactive oxygen species. Proceedings of the National Academy of Sciences of the United States of America 110: 19408-19413.

4. Fujino T, Ide T, Yoshida M, Onitsuka K, Tanaka A, et al. (2012) Recombinant mitochondrial transcription factor A protein inhibits nuclear factor of activated T cells signaling and attenuates pathological hypertrophy of cardiac myocytes. Mitochondrion 12: 449-458.
